# Improved Standardization of Flow Cytometry Diagnostic Screening of Primary Immunodeficiency by Software-Based Automated Gating

**DOI:** 10.3389/fimmu.2020.584646

**Published:** 2020-11-02

**Authors:** Eleni Linskens, Annieck M. Diks, Jana Neirinck, Martín Perez-Andres, Emilie De Maertelaere, Magdalena A. Berkowska, Tessa Kerre, Mattias Hofmans, Alberto Orfao, Jacques J. M. van Dongen, Filomeen Haerynck, Jan Philippé, Carolien Bonroy

**Affiliations:** ^1^ Department of Laboratory Medicine, Ghent University Hospital, Ghent, Belgium; ^2^ Department of Immunohematology and Blood Transfusion, Leiden University Medical Center, Leiden, Netherlands; ^3^ Department of Diagnostic Science, Ghent University, Ghent, Belgium; ^4^ Cancer Research Centre (IBMCC, USAL-CSIC; CIBERONC CB16/12/00400), Department of Medicine and Cytometry Service (NUCLEUS Research Support Platform), Institute for Biomedical Research of Salamanca (IBSAL), University of Salamanca (USAL), Salamanca, Spain; ^5^ Translational and Clinical Research Program, Centro de Investigación del Cáncer and Instituto de Biología Molecular y Celular del Cáncer, Consejo Superior de Investigaciones Científicas (CSIC)-University of Salamanca (USAL), Department of Medicine, IBSAL and CIBERONC, University of Salamanca, Salamanca, Spain; ^6^ Department of Hematology, Ghent University Hospital, Ghent, Belgium; ^7^ Department of Pediatric Pulmonology and Immunology and PID Research Laboratory, Ghent University Hospital, Ghent, Belgium

**Keywords:** ****flow cytometry, immunophenotyping, primary immunodeficiencies, automated gating, standardization, EuroFlow

## Abstract

**Background:**

Multiparameter flow cytometry (FC) is essential in the diagnostic work-up and classification of primary immunodeficiency (PIDs). The EuroFlow PID Orientation tube (PIDOT) allows identification of all main lymphocyte subpopulations in blood. To standardize data analysis, tools for Automated Gating and Identification (AG&I) of the informative cell populations, were developed by EuroFlow. Here, we evaluated the contribution of these innovative AG&I tools to the standardization of FC in the diagnostic work-up of PID, by comparing AG&I against expert-based (EuroFlow-standardized) Manual Gating (MG) strategy, and its impact on the reproducibility and clinical interpretation of results.

**Methods:**

FC data files from 44 patients (13 CVID, 12 PID, 19 non-PID) and 26 healthy donor (HD) blood samples stained with PIDOT were analyzed in parallel by MG and AG&I, using Infinicyt™ software (Cytognos). For comparison, percentage differences in absolute cell counts/µL were calculated for each lymphocyte subpopulation. Data files showing differences >20% were checked for their potential clinical relevance, based on age-matched percentile (p5-p95) reference ranges. In parallel, intra- and inter-observer reproducibility of MG vs AG&I were evaluated in a subset of 12 samples.

**Results:**

The AG&I approach was able to identify the vast majority of lymphoid events (>99%), associated with a significantly higher intra- and inter-observer reproducibility compared to MG. For most HD (83%) and patient (68%) samples, a high degree of agreement (<20% numerical differences in absolute cell counts/µL) was obtained between MG and the AG&I module. This translated into a minimal impact (<5% of observations) on the final clinical interpretation. In all except three samples, extended expert revision of the AG&I approach revealed no error. In the three remaining samples aberrant maturation and/or abnormal marker expression profiles were seen leading in all three cases to numerical alarms by AG&I.

**Conclusion:**

Altogether, our results indicate that replacement of MG by the AG&I module would be associated with a greater reproducibility and robustness of results in the diagnostic work-up of patients suspected of PID. However, expert revision of the results of AG&I of PIDOT data still remains necessary in samples with numerical alterations and aberrant B- and T-cell maturation and/or marker expression profiles.

## Introduction

Primary immunodeficiency (PIDs) comprises a clinically heterogeneous group of rare disorders with defects in the innate and/or adaptive immune system. Due to the dysfunctional immune system, patients can suffer from a wide variety of clinical manifestations, including severe, recurrent and opportunistic infections, auto-inflammation and auto-immunity (1–3). Since delayed diagnosis causes higher morbidity and mortality, fast and efficient PID diagnosis, classification and risk assessment is critically important.

Multicolor flow cytometric (FC) immunophenotyping has become a key tool in the diagnostic work-up and classification of PID (1, 3–7). FC has the advantage of providing fast, widely accessible and relatively low-cost diagnostic screening (8) based on a wide range of assays devoted to immunophenotypic identification and enumeration of specific (sub)populations of blood lymphocytes (e.g. B, T and NK cell subsets), quantitative evaluation of disease-associated protein expression profiles—e.g. Wiskott-Aldrich syndrome protein screen (WASP), CD40/CD40Ligand expression for hyper IgM syndromes-, functional assays (e.g. lymphocyte/T-cell proliferation) and analysis of specific signaling pathways (e.g. phosphorylation of STAT proteins) (2, 7–9).

Despite the clinical relevance of FC in the diagnosis and classification of PID, standardization of specific FC assays across distinct laboratories still remains a challenge. Thus, most published FC data on PID are limited to single center datasets which may not be directly applicable in other centers. Generation of reproducible and comparable data in multicenter settings is required for (inter)national data exchange and integration, creation of larger datasets of patient samples and better identification and definition of the altered immunophenotypic patterns associated with specific PID diagnostic categories.

In the past years, the EuroFlow consortium developed a diagnostic algorithm together with fully standardized antibody combinations (preferably used as dried format reagent mixes) (10) and analytical FC procedures for the diagnostic screening and classification of PID of the lymphoid system (6). In the proposed EuroFlow approach, the PID orientation tube (PIDOT) plays a central role in case of suspicion of PID, as recently validated in a selected cohort of genetically defined PID patients (4). Overall, PIDOT allows unequivocal and reproducible identification of >20 different leucocyte populations (including 15 T, B and NK lymphoid subpopulations) in blood, when more than a million cells are evaluated. Interpretation of such data using the classical (2-dimensional-based) expert-guided Manual Gating (MG) approaches (i.e. Boolean gating strategies) is time-consuming and highly subjective, as it relies on the operator’s gating decisions, knowledge and expertise. Thereby, MG strategies may result in disturbing levels of variability and more limited reproducibility of FC data analysis, depending on the knowledge and experience of each individual expert (11–14). In order to avoid such variability introduced during data analysis, the EuroFlow consortium has developed innovative Automated Gating and Identification (AG&I) approaches and software tools (14), which can be directly applied to the analysis of FC standard data files of blood samples stained with the PIDOT. This tool is based on the combined use of clustering algorithms and big data-based classification approaches, including direct comparison of individual clusters of events per interrogated sample against i) a fully annotated database of FC data files from healthy individuals stained according to the same standard operating procedures (SOPs), and ii) reference values based on a large dataset of hundreds of age-matched healthy donors that includes samples from controls between 0 days (neonatal) and 89 year-old subjects (4, 14).

In this study, we evaluated the contribution of the AG&I module available in the Infinicyt software (Cytognos Sl, Salamanca, Spain), in combination with the PIDOT antibody panel and database, for an increased reproducibility and standardization of multiparameter FC analysis of lymphocyte populations in blood of patients suspected of PID, compared to the classical EuroFlow-standardized MG strategy. In parallel, we also evaluated the potential impact of the new AG&I tool vs. the classical MG approach on the clinical interpretation of PIDOT results.

## Materials and Methods

### Sample Collection

FC PIDOT data files of peripheral blood (PB) samples from 44 patients, collected in a routine context of PID suspicion at the Hematology Laboratory of the Ghent University Hospital between November 2016 and March 2018, were included in this study. From these patients, 13 were diagnosed with common variable immunodeficiency (CVID) according to the ESID criteria (9) (M/F ratio: 8/5; age range: 7–67y), and 12 with other PID (M/F ratio: 5/7; age range: 1–12y; two patients with Shwachman–Bodian–Diamond Syndrome [SBDS]; two with KMT2A deficiency; two with myeloperoxidase (MPO) deficiency; one patient with tumor necrosis factor receptor-associated periodic syndrome [TRAPS]; one with KMT2D deficiency; one with adenosine deaminase (ADA) deficiency; one with IRAK4 deficiency and two with mannose-binding lectin (MBL) deficiency); the other 19 cases corresponded to non-PID patients with PID suspicion at time of sampling in whom the diagnosis of PID was ruled out ([non PID]; M/F ratio: 12/7; age range: 11m–50y). In addition, FC PIDOT data files from 26 healthy donors (HD) (M/F ratio: 10/16; age range: 20–58y) collected at Leiden University Medical Center (n=16) and at the Hematology Laboratory of the Ghent University Hospital (n=10) were included in the study (15). The study was approved by the local ethics committee of the Ghent University Hospital, Belgium (approval 2016/1137). Informed consent from the adult healthy volunteers included in the study was obtained at the time of blood sampling at the participating centers (15).

### Staining Procedures, Instrument Set-Up, and Data Acquisition

PB samples were collected in BD Vacutainer tubes containing K_2_EDTA (Becton/Dickinson, San Jose, CA). For each sample, a white blood cell (WBC) count was determined on a Sysmex XP-300 hematology analyzer (Sysmex Corporation, Kobe, Japan). For the immunophenotypic studies non-nucleated red cells were lysed prior to staining, strictly following the EuroFlow bulk lyse SOP (available at www.EuroFlow.org), as described elsewhere (16, 17). Subsequently, a stain- wash protocol was performed. Thus, the remaining cell pellet in a volume of 100µL, was stained for 30 minutes in the dark (room temperature [RT]) with the EuroFlow PIDOT monoclonal antibody combination, as previously described (liquid format) (4). Afterwards, 2mL of BD FACS™ Lysing solution –Becton/Dickinson Biosciences (BD)- diluted 1/10 (v/v) in distilled water, was added and the cell suspension was incubated for another 10 minutes at RT in the dark. Afterwards, cells were washed and the cell pellet was re-suspended in 300µL of washing buffer. Staining and data acquisition of all samples were performed within 24h after blood collection.

Data were acquired on BD FACSCanto™ II flow cytometers (BD) at the collection sites. In both centers, instrument settings and data acquisition were performed according to the EuroFlow guidelines available at www.EuroFlow.org (11). Standard instrument settings were monitored by BD™ Cytometer Setup and Tracking (CS&T) beads (BD) and eight-peak Rainbow bead calibration particles (Spherotech, Lake Forest, IL). For each sample at least 10^6^ total events were acquired at low-medium speed. Subsequently, data were exported as an FC standard-file for further analysis. As per the EuroFlow standard instrument setting and calibration SOPs, further manual compensation for optimization of measurements of individual samples was not required.

### Data Analysis

All FC standard data files were analyzed using Infinicyt™ Software (version 2.0.1b, Cytognos SL, Salamanca, Spain) both manually (MG strategy) and automatically (AG&I) using the Infinicyt™ AG&I module and the EuroFlow PIDOT database, with a special focus on the lymphoid populations.

MG was based on the previously published EuroFlow PIDOT guidelines (4, 6). Briefly, gating of the lymphoid populations was performed after excluding debris and cell doublets based on sideward light scatter area (SSC-A)/forward light scatter area (FSC-A) and FSC Height (FSC-H)/FSC-A bivariate dotplots, respectively. B-cells were identified based on their unique CD45^hi^ CD19^+^ CD3^-^ CD45RA^+^ phenotype and FSC^lo^ SSC^lo^ characteristics. Further identification of B-cell subpopulations was based on the levels of expression of CD27, IgM and IgD. In turn, T-cells were identified based on a CD45^hi^ CD3^+^ and FSC^lo^ SSC^lo^ phenotype. After gating TCRγδ^+^ T-cells, the CD4^+^, CD8^+^ and CD4^-^ CD8^-^ TCRγδ^-^ T-cell subpopulations were identified. Subsequently, the distinct maturation-associated subsets of CD4^+^ and CD8^+^ TCRγδ^-^ T-cell subpopulations were further identified based on their unique levels of expression of CD27 and CD45RA. Finally, NK-cells were defined as CD45^hi^CD19^-^CD3^-^CD16&CD56^hi^CD45RA^lo to +^ FSC-A^lo^ SSC-A^lo^ cells. More details on the MG strategy used for the identification of the lymphoid populations are provided in **Table 1**.

**Table 1:** Phenotypic features used in the Manual Gating (MG) strategy for the identification of lymphoid populations in blood according to the EuroFlow guidelines for analysis of blood samples stained with PIDOT.

Population	Gating strategy ^(1)^
B-cells	FSC^lo^ SSC^lo^CD45^hi^CD19^+^CD3^-^CD45RA^+^
Pre-germinal center B-cells	CD27^-^IgD^+^IgM^+^
Post-germinal center B-cells/plasmacells (MBC/PC)	
Unswitched MBC/PC ^(2)^	IgD^+^IgM^+^CD27 ^+^
Switched MBC/PC ^(2)^	IgD^-^IgM^-^CD27^- to +^
IgD^+^IgM^-^ post-GC	IgD^+^IgM^-^CD27^+^
T-cells	FSC^lo^ SSC^lo^CD45^hi^CD3^+^CD19^-^CD16&CD56^- to lo^
TCRγδ^+^ T-cells	TCRγδ ^+^CD4^-^CD8^- to lo^
TCRγδ^-^ CD4^-^CD8^-^ T-cells	TCRγδ ^-^CD4^-^CD8^- to lo^
CD4^+^ T-cells	TCRγδ ^-^CD4^+^CD8^-^
CD4^+^ naive T-cells	CD27^+^CD45RA^+^
CD4^+^ central memory T-cells	CD27^+^CD45RA^-^
CD4^+^ effector memory T-cells	CD27^-^CD45RA^-^
CD4^+^ terminal effector T-cells	CD27^-^CD45RA^+^
CD8^+^ T-cells	TCRγδ ^-^CD4^-^CD8^+^
CD8^+^ naive T-cells	CD27^+^CD45RA^+^
CD8^+^ central memory T-cells	CD27^+^CD45RA^-^
CD8^+^ effector memory T-cells	CD27^-^CD45RA^-^
CD8^+^ effector CD27+ T-cells	CD27^lo^CD45RA^+^
CD8^+^ terminal effector T-cells	CD27^-^CD45RA^+^
CD4^+^CD8^+^ T-cells	TCRγδ ^-^CD4^+^CD8^+^
Natural Killer cells	SSC-A^lo^FSC-A^lo^CD45^hi^CD19^-^CD3^-^CD16&CD56^hi^CD45RA^lo to +^

^(1)^In addition to the classical two-dimensional gating based on the listed markers, automatic population separator (APS) plots were used for fine-tune the gating of the listed cell populations as described elsewhere (4); ^(2)^Most MBC/PC, but not all, are CD27^+^.

In parallel with the MG strategy, all FC standard data files were also analyzed with the AG&I module of Infinicyt™. The AG&I module compares each FC standard data file with a reference database of healthy controls using the automated gating and classification algorithms, as previously described in detail (18, 19). This automated analysis included a first clustering step of all individual events in the data file, followed in a second step by classification of the resulting clusters of events into the cell populations identified *a priori* in the database, according to their characteristics in the multidimensional space generated by all the parameters evaluated. Input of patient’s age and WBC counts was required before the automated process could be started. During automated data processing, most events are automatically assigned to the different cell populations with only a few remaining unassigned clusters of events (= “checks”). For these latter groups of alarmed events, the AG&I module proposes one or more populations to which they might correspond, but definitive assignment to a given cell population must be done manually by an expert. Once the alarmed events have been checked by the expert, the software provides an automated report indicating the normal range in age-matched controls, with a “remark” for each cell population with values out of the normal range, using previously published reference values (4, 6). In this study, those events automatically assigned to a given cell population were not re-evaluated or re-classified manually to mimic the optimal routine situation, unless stated otherwise. Following the automated gating process, the percentage of each cell population from both its parent population and all WBC, was automatically calculated, recorded and stored by the Infinicyt™ software. Absolute cell counts/µL were calculated according to the white blood cell count (dual platform) as follows:

$$\frac{Relative\;WBC\;(\%)}{100}x\;WBC\;/\mu L=absolute\;count/\mu L$$

#### Comparison Between the MG Strategy and the AG&I Approach

Absolute cell counts/µL obtained with the MG and AG&I strategies were compared for each cell population. The percentage difference between the counts for each cell population obtained with the two strategies was calculated by the following formula:

$$\frac{(absolute\;count/\mu L)\;MG-(absolute\;count/\mu L)\;AG\&I}{(absolute\;count/\mu L)MG}x100$$

According to the International Standard EN ISO 15189 (20), the two gating methods were considered to be equivalent whenever the percent difference was <20%. Nevertheless, for the less abundant subpopulations with relatively wide reference intervals, the application of the <20% difference criterion may be clinically irrelevant. Because of this, for all lymphoid subpopulations with >20% differences we applied an additional criterion that relied on the impact on the final clinical interpretation, based on comparison of each of the two values against age-matched reference percentile (p5–p95) ranges as determined on a group of 250 HD (4). Differences in interpretation of the results after application of these age-matched reference values were considered as ‘clinically relevant’ and triggered a more detailed revision of both the AG&I (including a revision of the automatically assigned events whenever necessary) and MG analyses.

#### Intra- and Inter-Observer Reproducibility

Twelve samples (three samples from each patient group and the HD group) out of the 70 samples analyzed were randomly chosen to document the impact of the use of the AG&I software tools vs MG, on intra-observer and inter-observer reproducibility of data analysis.

For evaluation of intra-observer reproducibility, MG and AG&I were performed five times by the same observer (EL) on those 12 samples selected as described above. For inter-observer reproducibility, MG and AG&I were performed on the same 12 samples by five different observers (EL, MH, CB, PB, JDW). All five observers were trained individuals with strong expertise in gating the EuroFlow PIDOT tube and (routine) users of Infinicyt™. Standard deviation (SD) and coefficient of variation (CV) values were calculated for each cell population as obtained by both gating strategies.

### Statistical Methods

For comparison of the analysis strategies, Spearman rank correlation was used. For comparison between groups for continuous variables, the Chi-squared test with the Yates’ correction for continuity, was applied. Statistical comparison of the CVs was performed by the variance ratio F-test. Two-sided p-values <0.05 were considered to be associated with statistical significance. In case of multiple testing, the Bonferroni correction was applied. Statistical evaluation was performed using MedCalc Statistical Software (version 15.6.1; MedCalc Software bvba, Ostend, Belgium) and GraphPad Prism (version 5.04 for Windows; GraphPad Software, San Diego, CA).

## Results

### Comparison Between MG and AG&I Approaches on Healthy Donor Blood Samples

When considering all observations for the HD samples (n=520 observations; 20 cell populations in 26 samples), the vast majority of the events in the HD FC standard data files—median of 99.85% (range: 99.30–99.95%)—that corresponded to lymphoid cells were classified into one of the lymphocyte populations of the database with the AG&I module. In contrast, for a minor fraction of events—median 0.15% (range 0.0–0.70%)—the AG&I module induced an alarm due to phenotypic deviations from the reference populations in the PIDOT database, and required revision by an expert.

Differences greater than 20% on absolute cell counts (/µL) as calculated *via* MG vs AG&I were observed in 17% of all HD observations. An overview of these differences per cell population is shown in **Table 2**. Briefly, no differences >20% between both analytical strategies (MG and AG&I) were observed for 9/20 lymphoid populations (i.e. total lymphocytes, B-cells, pre-GC B-cells, T-cells, CD4^+^ T-cells, CD4^+^ naïve and central memory T-cells, CD8^+^ T-cells, and TCRγδ^+^ T-cells). In turn, a limited number of HD samples showed >20% differences between MG and AG&I counts for NK-cells (n=3/26), unswitched memory B-cells/plasma cells (MBC/PC) (n=8/26), switched MBC/PC (n=2/26), CD4^+^ effector memory T-cells (n= 2/26), CD8^+^ naive T-cells (n=2/26), CD8^+^ central memory T-cells (n=5/26) and CD4^-^CD8^-^ TCRγδ^-^ T-cells (n=6/26). In contrast, differences were more frequently observed [60% of the observations (n=62/104)] for other less abundant T-cell subpopulations [CD4^+^ terminal differentiated (TD) T-cells (n=20/26), CD8^+^ effector memory T-cells (n=11/26), CD8^+^ TD27^+^ T-cells (n=19/26) and CD8^+^ TD T-cells], as might have been expected for these populations which typically represent <1% of all WBC. Of note, CD4^+^CD8^+^ double-positive T-cells were not assigned as a separate population with the AG&I module. Spearman rank correlation coefficients for cell populations mandatory for PID screening and classification according to the ESID criteria (i.e. total lymphocytes, total B-cells, pre-GC B-cells, unswitched and switched MBC/PC, total T-cells, CD4^+^ and CD8^+^ T-cells, CD4^+^ and CD8^+^ naïve and central memory T-cells, TCRγδ^+^ and TCRγδ^-^ CD4^-^CD8^-^ T-cells, NK-cells) are shown in **Table 3**. For healthy donor samples correlation coefficients of >0.90 were obtained for most populations.

**Table 2:** Comparison of the Manual Gating (MG) strategy versus the AG&I module.

*Population *	n of samples (%) with > 20% numerical differences						n of samples (%) with clinically relevant differencesvs age-matched (p5 - p95) normal reference values					
	Total samplesn = 70	Patient samples n = 44	CVIDn = 13	PIDn = 12	Non PIDn = 19	HDn = 26	Total samplesn = 70	Patient samples n = 44	CVIDn = 13	PIDn = 12	Non PIDn = 19	HDn = 26
*Lymphocytes*	1 (1.4)	1 (2.3)	1 (7.7)	0 (0)	0 (0)	0 (0)	0 (0)	0 (0)	NA	NA	NA	0 (0)
*B-cells*	1 (1.5)	1 (3.8)	1 (7.7)	0 (0)	0 (0)	0 (0)	0 (0)	0 (0)	NA	NA	NA	0 (0)
*Pre-GC B-cells*	6 (8.6)	6 (14)	4 (31)	2 (17)	0 (0)	0 (0)	1 (1.4)	1 (2.3)	1 (7.7)	0 (0)	0 (0)	0 (0)
*Unswitched* *MBC/PC*	35 (50)	27 (61)	9 (69)	10 (83)	8 (42)	8 (31)	6 (8.6)	6 (14)	2 (15)	4 (33)	0 (0)	0 (0)
*Switched* *MBC/PC*	15 (21)	13 (30)	3 (23)	5 (42)	5 (26)	2 (7.7)	5 (7.1)	5 (11)	2 (15)	2 (17)	1 (5.3)	0 (0)
*T-cells*	1 (1.5)	1 (3.8)	1 (7.7)	0 (0)	0 (0)	0 (0)	0 (0)	0 (0)	NA	NA	NA	0 (0)
*CD4^+^ T-cells*	2 (2.9)	2 (4.5)	1 (7.7)	0 (0)	1 (5.3)	0 (0)	1 (1.4)	1 (2.3)	1 (7.7)	0 (0)	0 (0)	0 (0)
*CD4^+^ naive T-cells*	6 (8.6)	6 (14)	3 (23)	1 (8.3)	2 (11)	0 (0)	0 (0)	0 (0)	NA	NA	NA	0 (0)
*CD4^+^ central* *memory T-cells*	10 (14)	10 (23)	1 (7.7)	4 (33)	5 (26)	0 (0)	3 (4.3)	3 (6.8)	0 (0)	1 (8.3)	2 (11)	0 (0)
*CD4^+^ effector* *memory T-cells*	10 (14)	8 (18)	3 (23)	3 (25)	2 (11)	2 (7.7)	3 (4.3)	3 (6.8)	1 (7.7)	1 (8.3)	1 (5.3)	0 (0)
*CD4^+^ TD T-cells*	57 (81)	37 (84)	11 (85)	11 (92)	15 (79)	20 (77)	1 (1.4)	1 (2.3)	0 (0)	0 (0)	1 (5.3)	0 (0)
*CD8^+^ T-cells*	5 (7.1)	5 (11)	1 (7.7)	1 (8.3)	3 (16)	0 (0)	0 (0)	0 (0)	NA	NA	NA	0 (0)
*CD8^+^ naive T-cells*	11 (16)	9 (20)	5 (38)	1 (8.3)	3 (16)	2 (7.7)	2 (2.9)	2 (4.5)	0 (0)	0 (0)	2 (11)	0 (0)
*CD8^+^ central* *memory T-cells*	29 (41)	24 (55)	5 (38)	8 (67)	11 (58)	5 (19)	4 (5.7)	4 (9.1)	0 (0)	2 (17)	2 (11)	0 (0)
*CD8^+^ effector* *memory T-cells*	44 (63)	33 (75)	9 (69)	9 (75)	15 (79)	11 (42)	9 (13)	7 (16)	1 (7.7)	2 (17)	4 (21)	2 (7.7)
*CD8^+^ TD27^+^* *T-cells*	60 (86)	41 (93)	12 (92)	12 (100)	17 (89)	19 (73)	14 (20)	12 (27)	2 (15)	3 (25)	7 (37)	2 (7.7)
*CD8^+^ TD27^-^* *T-cells*	44 (63)	32 (73)	10 (77)	8 (67)	14 (74)	12 (46)	5 (7.1)	3 (6.8)	1 (7.7)	2 (17)	0 (0)	2 (7.7)
*CD4^-^CD8^-^ TCRγδ ^-^ T-cells*	20 (29)	14 (32)	4 (31)	5 (42)	5 (26)	6 (23)	6 (8.6)	6 (14)	4 (31)	1 (8.3)	1 (5.3)	0 (0)
*TCRγδ^+^ T-cells*	6 (8.6)	6 (14)	4 (31)	0 (0)	2 (11)	0 (0)	1 (1.4)	1 (2.3)	1 (7.7)	0 (0)	0 (0)	0 (0)
*NK-cells*	9 (13)	6 (14)	4 (31)	2 (17)	0 (0)	3 (12)	1 (1.4)	0 (0)	0 (0)	0 (0)	0 (0)	1 (3.8)
*Total number of observations with deviations on total [% on total]*	*372/1400* *[27]*	*282/880* *[32]*	*92/260* *[35]*	*81/240* *[34]*	*108/380* *[28]*	*90/520* *[17]*	*62/1400* *[4.4]*	*55/880* *[6.3]*	*16/260* *[6.2]*	*18/240* *[7.5]*	*21/380* *[5.5]*	*7/520* *[1.3]*

CVID, Common Variable Immunodeficiency Disorder patients; PID, Other PID patients; Non PID: patients with diseases other than PID; HD, healthy donors; Pre-GC-B-cells, pre-germinal center B-cells; TD, terminal differentiated; NK-Cells, natural killer cells; NA, not applicable.

**Table 3:** Correlation between absolute counts obtained by manual gating (MG) versus automated gating and identification (AG&I).

	Healthy donors	Patients	Total
Lymphocytes	0.996	0.994	0.994
B-cells	0.996	0.998	0.998
Pre-GC B-cells	0.997	0.992	0.995
Unswitched MBC/PC	0.955	0.845	0.877
Switched MBC/PC	0.983	0.963	0.956
T-cells	0.997	0.992	0.992
CD4^+^ T-cells	0.998	0.989	0.990
CD4^+^ naive T-cells	0.976	0.988	0.989
CD4^+^ central memory T-cells	0.960	0.933	0.923
CD8^+^ T-cells	0.995	0.980	0.991
CD8^+^ naive T-cells	0.987	0.978	0.983
CD8^+^ central memory T-cells	0.779	0.933	0.893
TCRγδ^+^ T-cells	0.999	0.925	0.942
TCRγδ^-^ CD4^-^ CD8^-^ T-cells	0.951	0.915	0.955
Natural killer cells	0.883	0.983	0.973

Results expressed as Spearman rank correlation coefficient values for those cell populations that are mandatory for PID screening and classification according to the ESID criteria. For all correlations p-vales < 0.001 were detected.

**Figure 1:**
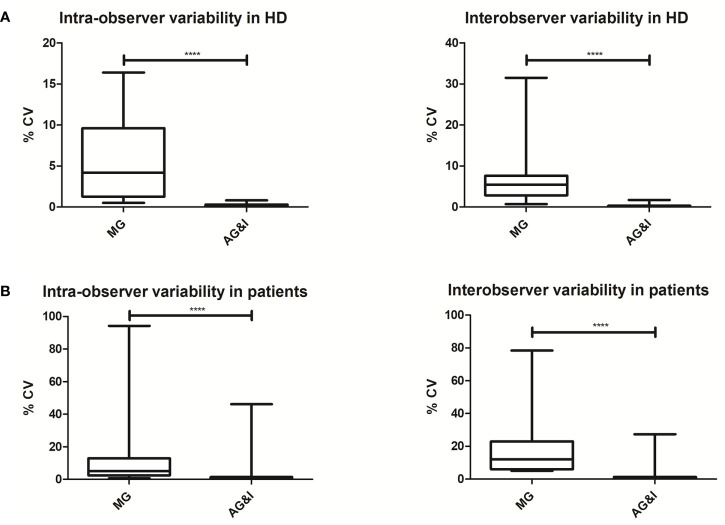
Intra- and inter-observer reproducibility of Manual Gating (MG) versus Automated Gating and Identification (AG&I). (**A**; top figure) Box-and-Whisker plots of CVs (%) for all lymphoid populations in HD blood samples. (**B**; bottom figure) Box-and-Whisker plots of CVs (%) for all lymphoid populations in patient samples. ****Statistically significant differences (P < 0.0001) based on the variance ratio F-test.

In order to further evaluate the impact of AG&I on both intra- and inter-observer reproducibility of data analysis on HD samples, SDs and median %CVs for MG and AG&I data, were compared (see **Figure 1A**). As a result, a lower overall median %CV was observed with the AG&I approach compared to MG (5.8 vs 0.2% for intra-observer and 8.0 vs 0.3% for inter-observer reproducibility, respectively). A more detailed analysis of the impact of AG&I for the individual cell populations is given in **Supplementary Table 1A**.

### Comparison of the MG Strategy Versus the AG&I Module on Samples of Patients Suspected of PID

A rather limited median percentage of checks (% of total events) was observed with the AG&I module (median 0.54%; range: 0.02–41.5%) for all observations recorded on the patient samples (n=880 observations for 20 cell populations in 44 samples) (**Supplementary Figure 1**).

Around one third (32%) of all patient samples showed >20% differences on the absolute cell counts (/µL) of at least one cell population as obtained with MG vs the AG&I module, with similar frequencies (p>0.05) in each of the three patient groups (CVID 35%, other PID 34%, and non-PID 28%). Despite this, differences >20% between both analytical strategies involving one or more of the major lymphocyte populations in blood (total lymphocytes, B-cells, T-cells, CD4^+^ and CD8^+^ T-cells) were restricted to a minority (<15%) of all patient samples. Thus, >20% differences were observed for total lymphocytes in 1/44 patients, B-cells in 1/44 cases, T-cells in 1/44, CD4^+^ T-cells in 2/44, and CD8^+^ T-cells in 5/44 cases. More than 20% differences between MG vs AG&I observed in the patient samples were mostly documented for the less abundant B- and T-cell subpopulations (counts <1% of all WBC). An overview of the results obtained per cell population is shown in **Table 2**. Spearman rank correlation coefficients for patient samples were calculated, showing correlation coefficients of >0.90 for most subset populations mandatory for PID screening and classification according to the ESID criteria (see **Table 3**).

The AG&I approach showed a greater intra- and inter-observer reproducibility than MG also on patient samples with lower median %CV (5.1 vs 0.4% for intra-observer and 12.1 vs. 0.6% for inter-observer reproducibility for AG&I vs MG, respectively) for all 20 lymphoid populations identified (see **Figure 1B** and **Supplementary Table 1B**).

### Impact of AG&I vs MG on Clinical Interpretation of Results

The possible impact of differences on clinical interpretation of cell counts obtained with the AG&I tool vs the MG strategy was evaluated by comparing each of the paired counts against age-matched reference percentile (p5–p95) ranges, assessed on a group of 250 HD (4). Data are summarized in **Table 2**. Overall, comparison of the results obtained with each of the two data analysis approaches against age-matched reference values, translated into different clinical interpretations for MG and AG&I in only 4.4% (n=62/1,400) of all paired observations [1.3% (n=7/520) for HD and 6% (n=55/880) for the patient samples].

Looking at the major lymphocyte populations (total lymphocytes, B-cells and T-cells) in both HD and patient samples, no difference in clinical interpretation was observed after evaluating the results against the age-matched p5–p95 reference values. The clinically relevant differences were also limited for the NK-cells, a different interpretation being restricted to a single HD sample (absolute NK-cell counts below p5 for MG while within the p5–p95 range for AG&I).

No differences in clinical interpretation related to the B-cell populations were observed in HD. In contrast, several differences in clinical interpretation were observed among the patients. Thus, clinically relevant differences in pre-germinal center B-cell counts were observed for one CVID patient sample for which differences against age-matched reference values were also detected for the memory B-cell populations as discussed in more detail below (Sample 1). Differences in clinical interpretation for unswitched and switched MBC/PC were mostly observed among CVID (n=2) and other PID samples (n=4; 2 KMT2A deficiencies, 1 IRAK4 deficiency, and 1 MBL). For the two KMT2A deficiency patients, who typically display a CVID-like phenotype with deviations in B-cell maturation and low absolute counts of different B-cell populations, the absolute counts obtained with MG were within age-matched reference ranges for both unswitched and switched MBC/PC, while AG&I provided decreased absolute counts for both memory B-cell populations below the p5. The IRAK4 deficiency sample and MBL deficiency sample (both conditions for which no lymphoid deviations are usually expected) also showed clinically relevant differences for the unswitched MBC/PC subpopulation, with abnormal values for the MG approach (below p5 for the IRAK4 deficiency and above p95 for the MBL deficiency), but normal absolute counts for all B-cell subpopulations when analyzed with the AG&I tool. In addition, another CVID patient sample (not Sample 1, see above), showed a clinically relevant difference in the absolute number of unswitched MBC/PC: decreased below p5 with MG while within the normal range with the AG&I approach. In another CVID sample, different clinical interpretation for switched MBC/PC was made with AG&I (absolute values below p5 as is expected for a CVID phenotype) and MG (absolute values within the p5–p95 range).

Differences in clinical interpretation related to CD4^+^ T-cell populations (including the less abundant CD4+ T cell populations) were also absent in HD and very limited in patient samples (0–7% of samples depending on the specific CD4^+^ T-cell population). No clinically relevant differences were found in HD for naive and central memory CD8^+^ T-cells, with only a few discrepancies in patient samples (between 5% and 9% of the samples depending on the specific CD8^+^ T-cell population). More differences in clinical interpretation (compared with age-matched normal p5–p95 reference ranges) were observed for the CD8^+^ effector T-cell populations (range: 7–27% of samples depending on the specific cell population). Despite all the above, detailed analysis of all clinically relevant differences observed for the distinct T-cell populations identified with PIDOT, showed no recurrent pattern (or cause) in all but one sample. This latter sample corresponded to a non-PID sample (Sample 2) which showed a combined pattern of clinically relevant differences in both CD4^+^ memory effector T-cells and CD4^-^CD8^-^ TCRγδ^-^ T-cells, as discussed in more detail below.

Different clinical interpretation for the TCRγδ^+^ and CD4^-^CD8^-^ TCRγδ^-^ T-cells were observed for a limited number of samples (1 and 6, respectively). One of these samples was already described above (Sample 2). In addition, one CVID sample (Sample 3) showed clinically relevant differences for both TCRγδ^+^ and CD4^-^CD8^-^ TCRγδ^-^ T-cells, triggering further investigations (see below). For the remaining four discrepant samples, no underlying cause could be identified to clarify the difference.

### Detailed Analyses of Clinically Relevant Discrepancies

**Figure 2:**
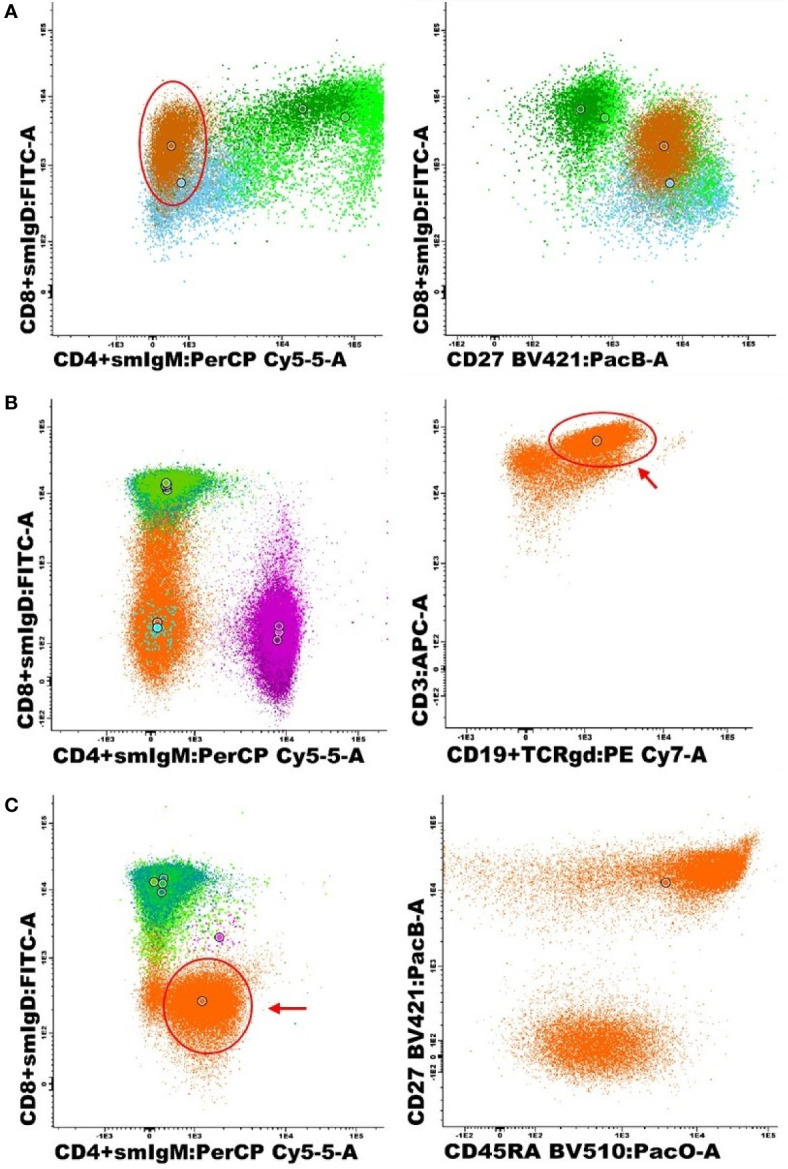
Representative (unchecked) AG&I bivariate dot-plots corresponding to the specific cell populations present in those 3 cases with altered phenotypes identified during detailed expert revision. **(A)** CVID sample (Sample 1) with a B-cell population showing abnormally dim expression of IgD on IgM-negative B-cells, classified by the AG&I tool as IgD^+^IgM^-^ MBC/PC (brown) with need for expert revision based on their aberrant expression pattern, in addition to pre-germinal center B-cells (dark green), unswitched MBC/PC (bright green) and switched MBC/PC (blue). **(B)** CVID sample with a large population of TCRγδ^+^ T-cells (see arrow) incorrectly assigned CD4^-^CD8^-^ TCRγδ^-^ events (orange) (sample 3) in addition to CD8^+^ T-cells (green), CD4^+^ T-cells (purple) and TCRγδ^+^ T-cells (blue). **(C)** Non PID patient with a large population of dim CD4^+^ events (see arrow) automatically classified as CD4^-^CD8^-^ TCRγδ^-^ T-cells (orange) (sample 2), in addition to CD8^+^ T-cells (green) and CD4^+^ T-cells (pink).

Detailed revision of both MG and AG&I data analysis was performed for all samples showing “clinically relevant” differences on the interpretation of the results obtained (once compared with age-matched reference values) for at least one lymphoid cell population (differences in 62 observations corresponding to 37 samples). In 34/37 samples (92%) AG&I results were confirmed during the expert review. In the remaining three samples (8%) corresponding to two CVID samples [Sample 1 and 3] and one non-PID patient sample [Sample 2] AG&I results were questionable. In more detail, in one of these two CVID samples [Sample 1], different absolute counts were observed for all B-cell populations identified by MG vs AG&I. Plots corresponding to the (unchecked) AG&I analysis for this sample are shown in **Figure 2A**. This was due to the fact that by AG&I a large proportion of the B-cells was automatically assigned to the memory IgD^+^IgM^-^ B-cell population. MG confirmed that phenotypically this population was indeed CD27^+^; however, its phenotype was not fully compatible with the classically high IgD expression (MFI of between 10^4^ – 10^5^ using the EuroFlow instrument settings in combination with the EuroFlow PIDOT reagents) on memory IgD^+^IgM^-^ B-cells, making their distinction from switched MBC/PC (CD27^+^/IgM^-^/IgD^-^) a challenge, also with MG that assigned them to switched MBC/PC. Despite this uncommon phenotype, AG&I analysis did not classify these events as ‘checks’. In the other two discordant samples (Samples 2 and 3) clinically relevant differences for the CD4^-^CD8^-^ TCRγδ^-^ T-cell population were observed and confirmed after revision of the AG&I data. In the latter CVID sample (Sample 3), a large TCRγδ^+^ population was automatically assigned to the CD4^-^CD8^-^ TCRγδ^-^ population by AG&I (unchecked AG&I plots shown in **Figure 2B**). In the non-PID sample (Sample 2), events classified by MG as CD4^+^ effector memory T-cells and CD4^+^ naive T-cells had been incorrectly classified by the AG&I module as CD4^-^CD8^-^ TCRγδ^-^ due to an abnormally low CD4-signal because of a technical (staining) issue (unchecked AG&I plots shown in **Figure 2C**). Despite all the above, these wrongly identified cell populations were systematically alarmed as “numerically altered” by the AG&I software tool, pointing out the need for review by the expert prior to final reporting.

## Discussion

Due to major technological advances in multiparameter FC, data analysis has become increasingly complex and time-consuming (13, 18, 21–23). This also implies higher variability and more subjectivity, as it is influenced by the observer’s expertise. Thus, the increased complexity associated with greater numbers of cells measured for an increasingly high number of parameters with the ability of identifying greater numbers of cell populations, has fostered the development of automated algorithms and tools for the analysis of complex multiparameter FC datasets (14, 18, 21–23). In this study, we evaluated the contribution of the EuroFlow AG&I module implemented in the Infinicyt software for analysis of PB samples stained with PIDOT in the standardization of the FC diagnostic work-up of PID.

Overall, our results showed that compared to MG, the use of the AG&I approach was associated with a significantly lower intra- and inter-observer variability of data analysis (and also interpretation) for all lymphoid populations identified with the PIDOT. In fact, the use of the AG&I tool systematically provided for most lymphoid populations identified a high intra- and inter-observer reproducibility with <20% CVs according to the EN ISO 15189 criterion applied in most European medical diagnostics laboratories (20). Altogether, these results indicate that replacement of MG by the AG&I module would be associated with a greater reproducibility and robustness of results.

In turn, a high degree of agreement (defined as <20% numerical differences in absolute cell counts/µL) was obtained between expert-based MG and the AG&I module, for most lymphocyte populations in both HD (83% of all observations) and routine diagnostic patient samples (68% of all observations), most discrepancies occurring for cell populations present at low (<1%) frequencies in blood. This translated into a minimal possible impact (4.4% of all observations, 6.3% of patient observations, 1.3% of HD observations) on the final clinical interpretation (e.g. normal vs increased or decreased cell counts) resulting from the comparison of the results obtained with each analytical approach with (p5–p95) age-matched reference values. Thereby, only a limited number of discordant observations between MG and AG&I was detected for those lymphoid populations that are mandatory for PID screening according to the ESID criteria (i.e. total lymphocytes, total B-cells, pre-GC B-cells, unswitched and switched MBC/PC, total T-cells, CD4^+^ and CD8^+^ T-cells, CD4^+^ and CD8^+^ naïve and central memory T-cells, TCRγδ^+^ and TCRγδ^-^ CD4^-^CD8^-^ T-cells, NK-cells). For those lymphoid populations, discrepant observations were restricted to 4.4% of patients observations (n=29/660 observations) and 0.2% of HD observations (n=1/390). This was due to the fact that most differences were observed for the less abundant T-cell populations (e.g. CD4^+^ and CD8^+^ effector memory and terminally differentiated T-cells), that are currently not considered in the (routine) diagnostic work-up of PID.

Looking into potential reasons for the discrepancies here reported between AG&I and MG, we identified three different variables to contribute to such differences: low absolute counts, suboptimal light scatter measurements and the use of single heterogeneously expressed markers for the distinction between two lymphoid populations. Thus, several of the less abundant T-cell subpopulations had low absolute counts for those samples with a >20% difference between AG&I and MG (Mann-Whitney U, p<0.05, see **Supplementary Data Table 2**). Besides, EuroFlow recommends well defined intervals for median FSC-A and/or SSC-A values for lymphocytes (median FSC-A of >50,000/<60,000 and median SSC-A of >11,000/<13,000). Here we observed that when these criteria were violated, an increased number of samples with >20% differences between AG&I and MG was found (see **Supplementary Data Tables 2** and **3**). For B-cells, the high number of >20% AG&I vs. MG differences observed for unswitched memory B-cells (and pre-GC B cells) could be related to the fact that these two B-cell populations are discriminated among them based upon a single marker/parameter with heterogeneous expression levels (i.e. CD27). This was confirmed by the significant lower delta MFI for CD27 between pre-GC and unswitched memory B-cells in the discrepant FC standard data file cases (median MFI values of 999 vs. 2441 arbitrary fluorescence channel values, Mann-Whitney U p=0.043) and vice versa (**Supplementary Data Figure 2**). Overall, these results indicate that AG&I is more reproducible and more accurate than MG in detecting abnormal values for individual cell populations (see below) due to technical issues, including those populations that are mainly discriminated based on a single, heterogeneously expressed marker (e.g. CD27 in unswitched memory B-cells vs pre-germinal center B-cells). Thus, these data indicate that strict adherence to the EuroFlow SOPs and criteria for instrument setup and calibration is mandatory, including a systematic check of the light scatter characteristics of lymphocytes for individual samples before final data storage, in parallel to careful evaluation of cell populations with low absolute count results.

Taken together, our results indicate that compared to conventional expert-based MG, routine use of the AG&I tool is associated with both a greater reproducibility of data analysis and a more robust interpretation of the numerical alterations detected for those lymphoid cell populations that are relevant in the diagnostic work-up of PID of the lymphoid system. However, for PID diagnosis, FC results should not be interpreted based on alterations involving single cell populations, but rather on the combination of altered patterns that typically affect >1 cell population within a sample.

As indicated above, numerical clinically relevant differences between AG&I and MG were more frequently observed among the patient samples than in HD samples. These results might be due to the fact that abnormal B- and T-cell maturation patterns and/or marker expression profiles are virtually restricted to patient samples and such altered profiles frequently represent a challenge during data analysis, even when MG is performed by an experienced operator. Indeed, clinically relevant differences within the B-cell subpopulations were found in several PID patients included in this study. For these PID patient-associated differences, it remains difficult to define which gating approach is correct due to the lack of a reference standard and the limited number of patients analyzed. Nevertheless, our results suggest a more accurate gating of B-cell populations using AG&I vs MG, since using the AG&I approach, B-cell alterations were only detected in the KMT2A-deficient patients, which are expected to have a CVID-like phenotype (24), but not in the IRAK4-deficient and MBL-deficient patients for whom no lymphoid deviations are usually expected (2, 9). Altogether, these findings underline the need for robust reference databases of PIDOT-stained blood samples from well-defined PID patients, in addition to normal HD blood, for unequivocal and accurate classification of cell events in PID suspicious patient blood samples, ideally generated in multi-center settings, as initiated by the EuroFlow PID consortium (4, 6, 13, 14, 25). Moreover, the availability of reference images of PID patients to the database might also contribute to a better classification of the more challenging cell populations and cases.

Despite all the above, the AG&I approach used here, based on the PIDOT database composed of HD blood samples stained with PIDOT at multiple centers, separately classifies all clusters of events that show phenotypic deviations from normal as groups of events that need to be checked by an expert. For HD samples, only a minor proportion of all events contained in the individual FC data files (<1% events) were classified as “checks”, i.e. events mimicking lymphoid cells that required expert revision following the AG&I classification tool. This observation is in line with previously published data on HD blood samples stained with other EuroFlow antibody combinations, that typically showed <2% checks of the total events (14). In contrast, some cell populations which are either absent or present at very low frequency in normal blood, such as activated B- and T-cells, might require expert revision, particularly for patient samples, as confirmed here.

Optimal use of the AG&I module for PIDOT would imply that in a first step, only unclassified clusters of events should be checked by an expert. Subsequently, all cell populations that carry phenotypic and/or numerical alarm should be revised, as done in this study for 37 samples that showed >20% differences in the absolute cell counts obtained for at least one cell population with the AG&I approach vs MG, that led to a distinct clinical interpretation after comparison with p5–p95 age-matched reference ranges (normal vs altered cell population). In all except three of these 37 samples, extended expert revision of the AG&I gating approach revealed no error. In the three remaining samples aberrant maturation and/or abnormal marker expression profiles were seen which might have induced an arguable classification of specific cell populations by the AG&I tool leading in all three cases to numerical alarms (i.e. absolute counts of the corresponding cell populations falling outside age-matched normal reference ranges). Thus, in a routine clinical laboratory setting, these latter deviations would trigger expert revision of the immunophenotypic results prior to their integration with other laboratory data and clinical findings. Although the underlying reason for these phenotypic deviations could not be fully identified, they might be due, at least in part to technical issues related with the quality of staining with single liquid format reagents (e.g. CD4 staining in one case, IgD staining in another patient and TCRγδ staining in third patient). In order to limit the impact of reagent variability and minimize pipetting issues, EuroFlow encourages the use of the lyophilized format of the PIDOT reagents (10).

In summary, here we show that the AG&I tool contributes to the standardization of FC data analysis in the diagnostic work-up of PID suspected patients, mostly due to its improved reproducibility vs conventional expert-based MG approaches and a more robust definition of numerical alterations against age-matched reference ranges. However, expert revision of the results of AG&I of PIDOT data still remains necessary in samples with numerical alterations and aberrant B- and T-cell maturation and/or abnormal marker expression profiles. Importantly, diagnosis of PID requires integration of FC results with other laboratory data (e.g. serum immunoglobulin levels, functional assays, molecular diagnostics, vaccination response), clinical findings and clinical history (e.g. history of infections), according to both the ESID and IUIS criteria (2, 6, 9). Of note, this study specifically focused on the evaluation of the technical performance of the new AG&I module for analysis of PIDOT data in a rather limited cohort of healthy donor and patient samples. Therefore, the overall impact of the new AG&I approach on the final PID diagnosis still remains to be fully defined in larger patient cohorts that include a wide variety of PID patients, preferably in a multi-center setting.

## Data Availability Statement

The raw data supporting the conclusions of this article will be made available by the authors, without undue reservation.

## Ethics Statement

The studies involving human participants were reviewed and approved by the local ethics committee of the Ghent University Hospital, Belgium (approval 2016/1137). Written informed consent to participate in this study was provided by the participants’ legal guardian/next of kin.

## Author Contributions

EL, AD, JN, MP-A, EM, MB, MH, AO, JD, JP, and CB contributed to the conception and design of the study. EL, AD, JN, EM, MB, TK, MH, and FH performed data acquisition and data analysis. MP-A provided the reference values. EL, AO, MP, JP, and CB wrote the manuscript. All authors contributed to the article and approved the submitted version.

## Funding

The coordination and innovation processes of this study were supported by the EuroFlow Consortium. The EuroFlow Consortium received support from the FP6-2004-LIFESCIHEALTH-5 program of the European Commission (grant LSHB-CT-2006-018708) as Specific Targeted Research Project (STREP). The EuroFlow Consortium is part of the European Scientific Foundation for Hemato-Oncology (ESLHO), a Scientific Working Group (SWG) of the European Hematology Association (EHA). JN is granted by the Fund for Scientific Research, TBM Funding, Belgium (T000119N). This work was also supported by a grant of the Grand Challenges Program of VIB.

## Conflict of Interest

JD, MP-A, and AO each report being one of the inventors on the EuroFlow-owned patent PCT/NL 2015/050762 (Diagnosis of primary immunodeficiencies). The Infinicyt software is based on intellectual property (IP) of the EuroFlow laboratories (University of Salamanca in Spain and Federal University of Rio de Janeiro in Brazil) and the scientific input of other EuroFlow members. All above mentioned intellectual property and related patents are licensed to Cytognos (Salamanca, ES) and BD Biosciences (San José, CA), which companies pay royalties to the EuroFlow Consortium. These royalties are exclusively used for continuation of the EuroFlow collaboration and sustainability of the EuroFlow consortium. JD and AO report an Educational Services Agreement from BD Biosciences and a Scientific Advisory Agreement from Cytognos; all related fees and honoraria are for the involved university departments at Leiden University Medical Center and University of Salamanca.

The remaining authors declare that the research was conducted in the absence of any commercial or financial relationships that could be construed as a potential conflict of interest.
