# Supplementary material for: Improved Standardization of Flow Cytometry Diagnostic Screening of Primary Immunodeficiency by Software-Based Automated Gating
Source: Front Immunol. 2020 Nov 2;11:584646. doi: 10.3389/fimmu.2020.584646 (PMC7667243; doi:10.3389/fimmu.2020.584646)
Supplement: Supplementary file 1 [file DataSheet_1.pdf]

Supplementary Data

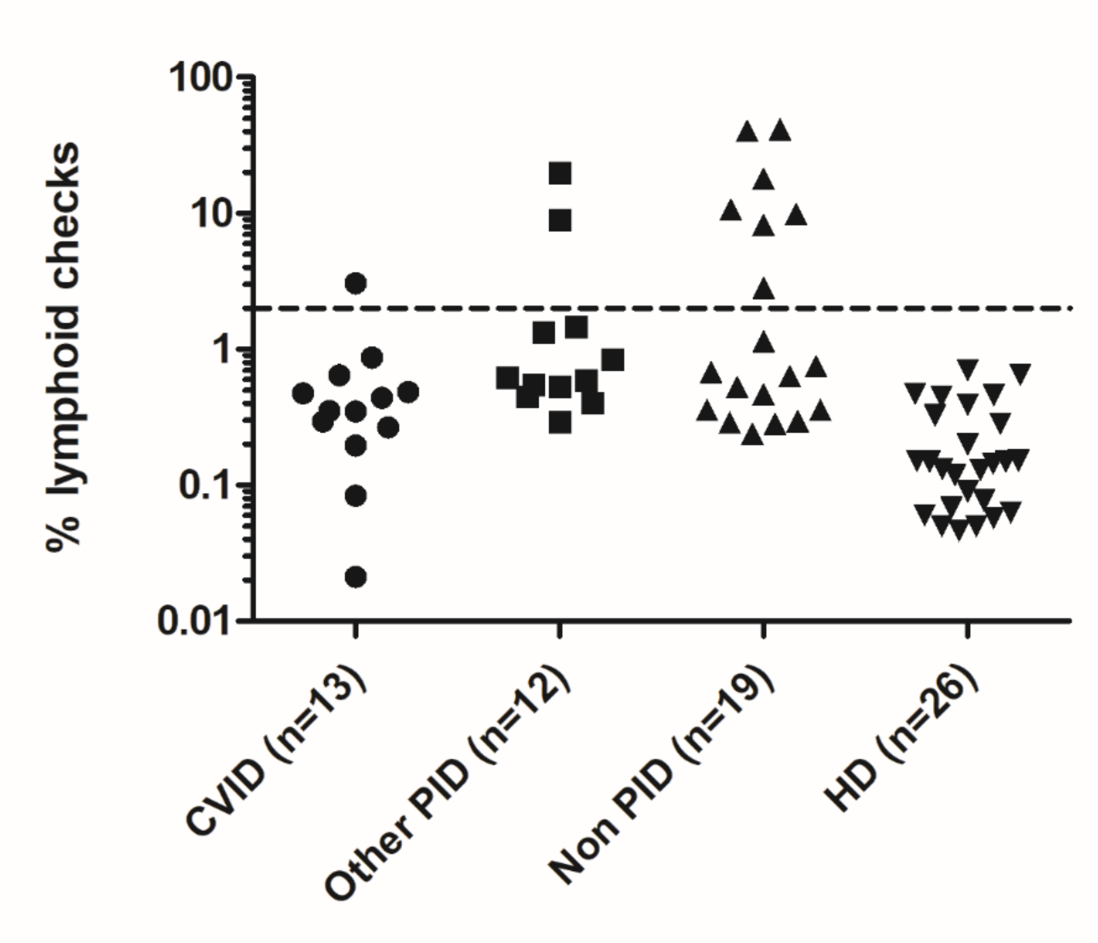

Supplementary Figure 1: Relative distribution of lymphoid checks (log scale) for each group of patients and healthy donors analysed in this study. **A dashed line** was set at 2% lymphoid checks, that according to previously published data on normal samples, corresponds to the acceptable percentage of checks from total events expected (14). **CVID:** Common variable immunodeficiency (n=13); **Other PID:** Primary immunodeficiency other than CVID (n=12); **Non PID:** Patients with diseases other than PID (n=19); **HD:** healthy donors (n=26)

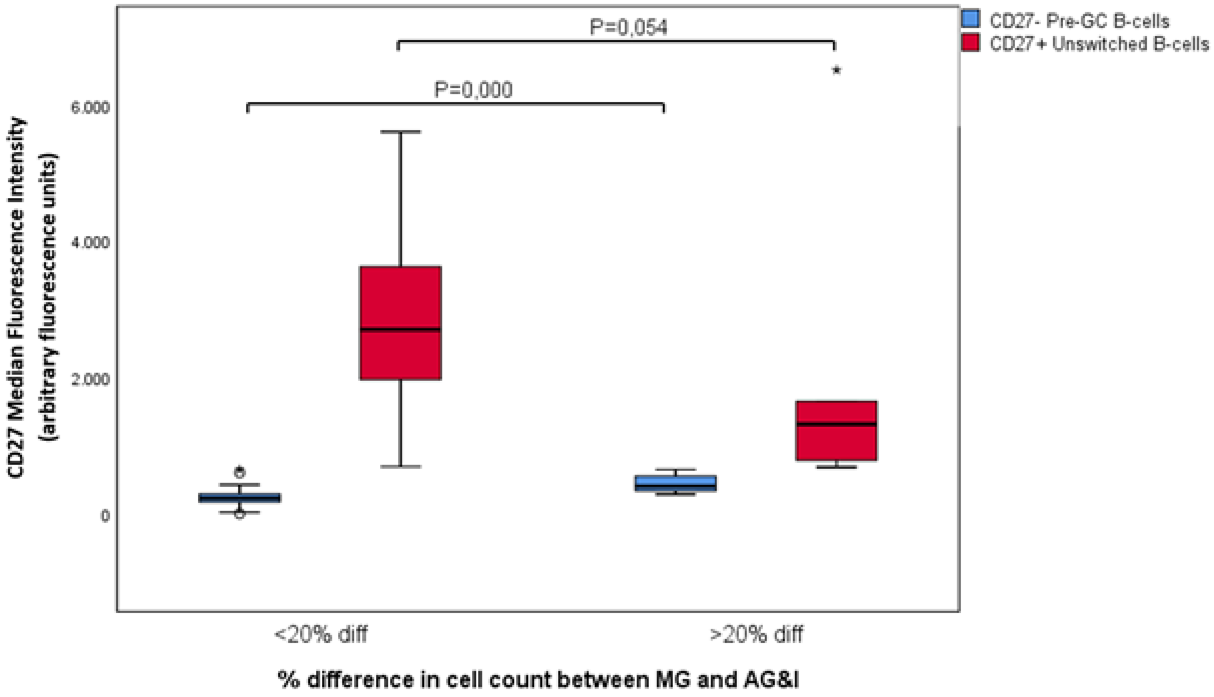

Supplementary Figure 2: Comparison between MFI values obtained for CD27 expression levels on **pre-GC B-cells** (blue boxes) and **unswitched memory B-cells** (red boxes) in samples classified based on the absence vs presence of >20% differences between AG&I and MG. Results show a better concordance between MG and AGI for those samples showing higher expression levels for CD27 on unswitched memory B cells. .

Supplementary Table 1.A: Intra- and inter-observer reproducibility of the MG vs AG&I approached in healthy donor samples (N=26).

	Intra-observer CV		Inter-observer CV	
	**MG**	**AG&I**	**MG**	**AG&I**
Lymphocytes	1.1	0.1*	1.4	0.2*
B-cells	9.9	0.2*	3.0	0.3*
Pre-GC B-cells	9.9	0.2*	2.8	0.2*
Unswitched MBC/PC	16.4	0.3*	6.9	0.3*
Switched MBC/PC	12.8	0.0*	7.0	0.4*
T-cells	0.6	0.1*	0.7	0.2*
$CD4^+$ T-cells	0.6	0.3	1.7	0.3*
$CD4^+$ naive T-cells	4.0	0.1*	4.8	0.3*
$CD4^+$ central memory T-cells	4.2	0.2*	4.8	0.2*
$CD4^+$ effector memory T-cells	3.6	0.8*	3.5	1.7
$CD4^+$ TD T-cells	5.9	0.0*	31.5	0.0*
$CD8^+$ T-cells	0.5	0.1	0.8	0.2
$CD8^+$ naive T-cells	2.5	0.1*	5.6	0.3*
$CD8^+$ central memory T-cells	1.8	0.0*	5.3	0.2*
$CD8^+$ effector memory T-cells	7.4	0.0*	5.6	0.0*
$CD8^+$ $TD27^+$ T-cells	11.7	0.5*	21.2	0.5*
$CD8^+$ TD T-cells	8.8	0.0*	6.5	0.0*
$TCR\gamma\delta^+$ T-cells	1.1	0.1*	7.8	0.2*
$TCR\gamma\delta^-$ $CD4^-$ $CD8^-$ T-cells	4.2	0.4*	18.1	0.0*
Natural killer cells	8.1	0.3*	14.7	0.4*

Results are expressed as median % CV for each individual lymphoid population.

Abbreviations: CV: coefficient of variation; MG: manual gating; AG&I: automated gating and identification; Pre-GC B-cells: pre-germinal center B-cells; MBC/PC: memory B-cells/plasma cells TD: terminal differentiated; NK-cells: natural killer cells

***** *Statistical comparisons among mean % CV values performed with the F-test; two-sided p-values <0.05 were considered to reflect statistically significant differences*

Supplementary Table 1.B: Intra- and inter-observer reproducibility of the MG vs AG&I approached in patient samples (N=44).

	**Intra-observer CV**		**Inter-observer CV**	
	**MG**	**AG&I**	**MG**	**AG&I**
Lymphocytes	1.0	0.1*	5.5	0.3*
B-cells	2.3	0.2*	6.0	0.3*
Pre-GC B-cells	2.6	0.3*	5.3	0.5*
Unswitched MBC/PC	22.0	2.8*	26.1	5.0*
Switched MBC/PC	13.2	1.8*	14.2	0.6*
T-cells	0.7	0.4*	6.3	0.5*
$CD4^+$ T-cells	0.8	0.2	5.4	0.4*
$CD4^+$ naive T-cells	4.0	0.4*	6.3	0.6*
$CD4^+$ central memory T-cells	4.3	0.5*	12.4	0.9*
$CD4^+$ effector memory T-cells	5.8	0.2*	14.2	0.6*
$CD4^+$ TD T-cells	94.2	46.1	78.4	27.4
$CD8^+$ T-cells	0.9	0.1*	5.0	0.3*
$CD8^+$ naive T-cells	2.9	0.3*	6.7	0.8*
$CD8^+$ central memory T-cells	6.6	0.5*	11.8	0.4*
$CD8^+$ effector memory T-cells	20.5	0.4*	28.5	0.3*
$CD8^+$ $TD27^+$ T-cells	15.7	9.1*	25.4	1.4*
$CD8^+$ TD T-cells	12.1	0.0*	24.4	0.2*
$TCR\gamma\delta^+$ T-cells	3.6	0.3*	13.6	1.2*
$TCR\gamma\delta^-$ $CD4^-$ $CD8^-$ T-cells	9.3	2.0*	10.6	9.5*
Natural killer cells	7.3	0.4*	18.9	2.1*

Results are expressed as mean % CV for each individual lymphoid population.

Abbreviations: CV: coefficient of variation; MG: manual gating; AG&I: automated gating and identification; Pre-GC B-cells: pre-germinal center B-cells; MBC/PC: memory B-cells/plasma cells TD: terminal differentiated; NK-cells: natural killer cells

** Statistical comparisons among mean % CV values performed with the F-test; two-sided p-values <0.05were considered to reflect statistically significant differences*

Supplementary Table 2: Differences in absolute cell counts and light scatter values obtained for samples that showed ≤20% vs >20 % differences in the final cell counts of ≥1 cell populations between AGI and MG

Mann-Whitney U testing performed for groups classified based on >20 % difference between AG&I and MG							
	**Frequency of samples with >20% differences**	**Absolute counts (/µL) for the specific population**		**Median SSC-A lymphocytes**		**Median FSC-A lymphocytes**	
		**Median (range) >20% difference**	**Median (range) <20% difference**	**Median (range) >20% difference**	**Median (range) <20% difference**	**Median (range) >20% difference**	**Median (range) <20% difference**
**B-cells**							
Pre-GC B-cells	9%	**27 (1.5 – 272)**	**209 (22 – 3789)***	57593 (33334 – 62758)	55104 (41983 – 73190)	11543 (7378 – 14645)	11948 (9007 – 16223)
Unswitched MBC/PC	50%	29 (0 – 254)	35 (1.5 – 242)	54693 (33334 – 67343)	55602 (41983 – 73190)	11538 (7378 – 14645)	12480 (9491 – 16223)
Switched MBC/PC	21%	23 (0.4 – 83)	37 (1.2 – 288)	52579 (42202 – 70676)	55329 (33334 – 73190)	11784 (9007 – 16166)	11955 (7378 – 16223)
**T-cells**							
$CD4^+$ naive T-cells	9%	314 (30 – 4788)	564 (41 – 3132)	49669 (41983 – 64294)	55388 (33334 – 73190)	10602 (9935 – 12239)	12048 (7378 – 16223)
$CD4^+$ central memory T-cells	14%	**256 (77 – 643)**	**397 (73 – 1548)****	**45592 (33334 – 57593)**	**56079 (41983 – 73190)***	**10287 (7378 – 12239)**	**12322 (9007 – 16223) ********
$CD4^+$ effector memory T-cells		47 (10 – 100)	33 (0 – 406)	57113 (41983 – 66296)	54939 (33334 – 73190)	12148 (10106 – 14465)	11893 (7378 – 16223)
$CD4^+$ TD T-cells	81%	0 (0 – 93)	0 (0 – 166)	55329 (33334 – 73190)	54446 (42202 – 67151)	11940 (7378 – 16223)	12455 (9007 – 15106)
$CD8^+$ naive T-cells	16%	**174 (6.9 – 399)**	**237 (19 – 1391) *****	54939 (46344 – 70676)	55329 (33334 – 73190)	11538 (9050 – 16166)	11955 (7378 – 16223)
$CD8^+$ central memory T-cells	41%	156 (15 – 1410)	156 (48 – 720)	52054 (33334 – 71787)	56425 (42272 – 73190)	16166 (7378 – 11538)	12480 (9050 – 16223)
$CD8^+$ effector memory T-cells	63%	6.2 (0 – 87)	11 (0.9 – 375)	53035 (41983 – 70676)	55782 (33334 – 73190)	11711 (9419 – 16166)	12256 (7378 – 16223)
$CD8^+$ $TD27^+$ T-cells	86%	7.4 (0 – 319)	19.1 (2.8 – 92)	**53491 (33334 – 73190)**	**59947 (46120 – 68585) *******	**11769 (7378 -16223)**	**13222 (9989 – 15253) *******
$CD8^+$ TD T-cells	63%	**11 (0 – 292)**	**27 (0 – 309) *******	52512 (33334 – 73190)	59163 (45080 – 71787)	**11742 (7378 – 16223)**	**12634 (9050 – 15253) *********
$TCR\gamma\delta^+$ T-cells	9%	28 (3 – 192)	86 (3 – 568)	53846 (41983 – 64914)	55269 (33334 – 73190)	11257 (10106 – 12946)	11997 (7378 – 16223)
$TCR\gamma\delta^-$ $CD4^-$ $CD8^-$ T-cells	29%	34 (2 – 320)	22 (2 - 102)	49001 (41983 – 70676)	55602 (33334 – 73190)	11848 (9007 – 16166)	11948 (7378 – 16223)

**p=0.001; **p=0.0042; ***p=0.040; ****p=0.008; *****p=0.02; ******p=0.002; *******p=0.03*

Abbreviations: MG: manual gating; AG&I: automated gating and identification; Pre-GC B-cells: pre-germinal center B-cells; MBC/PC: memory B-cells/plasmacells TD: terminal differentiated; NS: Not significant

Mann-Whitney U testing performed for groups classified based on >20 % difference between AG&I and MG; two-sided p-values <0.05 were considered to reflect statistically significant differences

Supplementary Table 3: Influence of the light scatter characteristics of lymphocytes on the percentage of cases with >20% differences in absolute cell counts obtained with MG vs AG&I.

	**EF criteria lymphocytes FSC >50,000**		**EF criteria lymphocytes SSC >11,000**		**EF criteria lymphocytes FSC >50,000 AND SSC>11,000**	
	Frequency of samples with > 20% MG vs. AG difference		Frequency of samples with > 20% MG vs. AG difference		Frequency of samples with > 20% MG vs. AG difference	
	Criteria fulfilled	Criteria not fulfilled	Criteria fulfilled	Criteria not fulfilled	Criteria fulfilled	Criteria not fulfilled
Lymphocytes	2%	0%	2%	0%	3%	0%
B-cells	2%	0%	2%	0%	3%	0%
Pre-GC B-cells	7%	9%	6%	14%	8%	10%
Unswitched MBC/PC	50%	49%	46%	59%	50%	50%
Switched MBC/PC	20%	26%	21%	23%	20%	23%
T-cells	2%	0%	2%	0%	3%	0%
$CD4^+$ T-cells	2%	4%	4%	0%	3%	3%
$CD4^+$ naive T-cells	7%	13%	4%	18%	3%	17%**
$CD4^+$ central memory T-cells	7%	30%*	8%	27%**	5%	27%*
$CD4^+$ effector memory T-cells	17%	9%	17%	9%	18%	10%
$CD4^+$ TD T-cells	83%	78%	83%	78%	80%	83%
$CD8^+$ T-cells	2%	17%**	4%	14%	3%	13%
$CD8^+$ naive T-cells	15%	17%	15%	18%	15%	17%
$CD8^+$ central memory T-cells	35%	57%	35%	55%	35%	50%
$CD8^+$ effector memory T-cells	52%	87%***	60%	68%	53%	77%****
$CD8^+$ $TD27^+$ T-cells	80%	96%	81%	96%	80%	83%
$CD8^+$ TD T-cells	54%	78%**	58%	73%	53%	77%****
$TCR\gamma\delta^+$ T-cells	9%	9%	6%	14%	5%	13%
$TCR\gamma\delta^-$ $CD4^-$ $CD8^-$ T-cells	22%	44%	27%	32%	23%	37%
Natural killer cells	15%	9%	13%	13%	15%	10%

*p=0.01; **p=0.04; ***p=0.004; ****p=0.03

Statistical comparisons among the frequencies of samples were performed with the Pearson's chi-squared test; two-sided p-values <0.05 were considered to reflect statistically significant differences

Abbreviations: EF: EuroFlow; FSC: Forward Scatter; SSC: Side Scatter; Pre-GC B-cells: pre-germinal center B-cells; MBC/PC: memory B-cells/plasma cells TD: terminal differentiated; NK-cells: natural killer cells
